# Learning from climate change news: Is the world on the same page?

**DOI:** 10.1371/journal.pone.0297644

**Published:** 2024-03-20

**Authors:** Stijn Eikelboom, Marc Esteve-Del-Valle, Malvina Nissim

**Affiliations:** 1 Department of Language Technology, Faculty of Arts, University of Groningen, Groningen, The Netherlands; 2 Research Centre for Media and Journalism Studies, Faculty of Arts, University of Groningen, Groningen, The Netherlands; 3 Center for Language and Cognition, Faculty of Arts, University of Groningen, Groningen, The Netherlands; Educational Testing Service (ETS), UNITED STATES

## Abstract

Climate change challenges countries around the world, and news media are key to the public’s awareness and perception of it. But how are news media approaching climate change across countries? With the problem of climate change and its solution being global, it is key to determine whether differences in climate change news reports exist and what they are across countries. This study employs supervised machine learning to uncover topical and terminological differences between newspaper articles on climate change. An original dataset of climate change articles is presented, originating from 7 newspapers and 3 countries across the world, and published in English during 26 Conference of the Parties (COP) meetings from the United Nations Framework Convention on Climate Change (UNFCC). Three aspects are used to discriminate between articles, being (1) countries, (2) political orientations, and (3) COP meetings. Our results reveal differences with regard to how newspaper articles approach climate change globally. Specifically, climate change-related terminology of left-oriented newspapers is more prevalent compared to their right-oriented counterparts. Also, over the years, newspapers’ climate change-related terminology has evolved to convey a greater sense of urgency.

## Introduction

Of the many challenges that mankind will face during the coming decades, it is likely that climate change will be one of the most pressing and demanding. Since the late 19th century, average global temperature has already risen by 1.1 degrees Celsius, which can largely be attributed to human-made emissions (climate.nasa.gov/evidence/). The sixth assessment report (AR6) of the Intergovernmental Panel on Climate Change (IPCC) points out that there is a more than 50% chance that global temperature rise will reach or surpass 1.5 degrees Celsius between 2021 and 2040 (ipcc.ch/report/sixth-assessment-report-cycle/). Therefore, climate change shall be high on the global agenda of scientific research, politics, and eventually every citizen.

An important means of calling attention to climate change is the (usually) yearly gathering of the *Conference of the Parties* (COP) from the *United Nations Framework Convention on Climate Change* (UNFCCC) (unfccc.int/process/bodies/supreme-bodies/conference-of-the-parties-cop). The convention strives to prevent “dangerous” human interference with the climate system (unfccc.int/process-and-meetings/the-convention/what-is-the-united-nations-framework-convention-on-climate-change) and the meetings are the chief platform for key decision-making. These have occurred 26 times between 1995 and 2019.

This study approaches climate change from a linguistic perspective. Language plays an important role in tackling the numerous effects of climate change. It facilitates precarious problem-solving during global political summits, it is used in scientific papers that must convince politicians to act, and it spreads the news to the general public [1–7].

Precisely the last context is considered crucial in this study. After all, behavioral change by every citizen is key to reducing greenhouse gas emissions. Politics and businesses must play a key role in facilitating this, but for citizens to really change their comings and goings, intrinsic conviction and motivation are essential.

News media are an important factor here, as they are the major source of climate information for most citizens [1], raising their understanding and shaping their perception [2], as well as impacting behaviour [8]. Politically, news media set the policy agenda [3, 4] and influence public debate [5, 9].

Moreover, climate change is fundamentally a global issue and not limited to country borders. It requires sustainable collaboration between countries and a consistent international approach. On the other hand, the audience of newspapers is very much constrained by country borders. To stay updated on national news, a national newspaper can be a key source but it usually provides a narrower and more local perspective. On top of that, newspapers have a tendency to report from a partisan political perspective [10]. As much as climate change is not constrained by geographical borders, it does not affect a single side of the political arena. However, this does not withhold politicians from vigorously debating the topic and related news becoming more and more politicized [11].

In short, news media are a major information source on climate change, in spite of their local and political character. These two notions together form the motivation for this study. With the problem of climate change and its solution being global, uncovering the differences in newspapers’ coverage of climate change can give insights into the underlying political processes. That makes it relevant to determine whether such variations exist and what they are.

This study aims to uncover differences between articles on climate change from English-language newspapers around the world. To deal with the lurking trade-off between thoroughness and scope, the potential of supervised machine learning is introduced. This allows to automatically analyze a large amount of data and consecutively reveal striking differences within it (see, for example, [12, 13]). Moreover, this data-driven approach can broaden the field, by revealing differences that might not be picked up by experts in manual analysis.

Specifically, the goal of this study is to detect topical and terminological differences between international newspaper articles on climate change. Since several aspects can lead to such differences, three characteristics are used to discriminate between the articles which thus become the prediction targets of the machine learning models. These are (1) the country of the newspaper, (2) the political orientation of the newspaper and (3) the COP meeting occurring during article publication. For their annual occurrence, the COP meetings are an ideal reflection of the moment of publication. Moreover, the meetings boost the publication of climate-related articles [14].

Machine learning is used to automatically conduct the analyses. Each aspect is separately analyzed through classification. Since COP meetings have a temporal nature, this aspect is also analyzed with regression. The resulting models are intended to gain more insight into key differences between articles. Hence, human-understandable features are used within statistical models. These are explicitly favored over more complex features and architectures that could improve performance, but are hard to interpret, such as word embeddings and neural networks.

Three research questions are answered:

1. *Are there major topical and terminological differences between the articles on climate change by newspapers from different countries?*
2. *Are there major topical and terminological differences between the articles on climate change by newspapers with different political orientations?*
3. *Are there major topical and terminological differences between the newspaper articles on climate change during different COP meetings?*

It is hypothesized that differences can be found between newspaper articles from different countries, particularly between countries from different continents [15, 16]. A larger contrast is expected to be seen between right and left-oriented newspapers, considering the issue seems to become ever more politicized [11]. Lastly, it is expected that differences exist between COP meetings as well, especially between editions that are further apart [14, 17].

## Previous work

Numerous studies have stressed the importance of mass media in communicating the concerns associated with climate change [15, 18–25]. Furthermore, news media can influence public concern about climate change by increasing levels of attention to it. Journalists, as Hase et al. contend [26], can present climate change as a more ubiquitous topic in the news by (a) stressing the sociological dimension of climate change and illustrating how humans are aware of, affected by, combating, or causing climate change [27], (b) focusing on how climate change affects public health [9] or (c) on activities that people can take [28], thereby encouraging public participation.

Climate change coverage can be distinguished by both domestic and global viewpoints on what causes it, who is affected by it, and how it can be addressed [29]. Several studies have been conducted to determine whether media coverage differs around the globe [30, 31]. According to [32], finding cross-national commonalities in the way climate change is reported is key because the existence of these commonalities may suggest the emergence of a public sphere as “an enduring structure that enables political debate and opinion formation for and with a global audience” (p. 689). However, research generally finds that discrepancies in climate change coverage persist [33]. These variations could be brought on by global factors that affect news media coverage, such as the political or economic contexts of various nations [34]. For instance, different national responsibilities for addressing climate change, politics, or vulnerabilities may have an impact on how much attention is paid to and how much is covered by the media [17].

According to an overview by Schmidt et al. [17], the majority of studies on climate change news focused on single countries, and only a few assessed differences between newspapers across nations. Also, among the studies assessing single and cross-country newspapers, manual content analysis seems to be the preferred method. As for the automated analysis of climate change-related news media, most of the relatively slim research on the topic relied on automated content analysis techniques [16, 17], while very little used machine learning [11, 14, 35]. Thus, the state of the art of the literature seems to indicate both the necessity to expand the cross-country analysis of climate change-related news media and the need to do so by exploring the use of machine learning techniques.

Certainly, machine learning can play a pivotal role in the analysis of vast climate change-related datasets. This is because machine learning enables the input of vast volumes of data into a computer algorithm, empowering the system to examine this data and generate data-driven recommendations and decisions solely based on the input information (see, for example, [6, 13, 36–39]). Consequently, the utilization of machine learning helps reduce human intervention in data manipulation and reduces the chance of introducing biases.

Grundmann and Scott [15] performed one of the first automated content analysis studies into the discourse of climate change over time. They used newspapers from France, Germany, the UK, and the USA. A corpus of 106.5 million words was created, by retrieving articles published between 2000 and 2010 from *Lexis Nexis*. The peaks and troughs of attention for climate change were visualized per country. Furthermore, keyword analysis such as *collocation analysis* was also employed. The goal of the research was to study which terminology each country favored to describe the concept of climate change. The authors also compared the attention paid to advocates and skeptics and analyzed the influence of *Climategate* (factcheck.org/2009/12/climategate/). The study revealed clear differences between the climate change terminology among the four countries. The study also showed that advocates were favored over skeptics in all countries, but more in Germany and the UK than in France and the USA. However, this study could methodologically only rely on manual detection of climate change-related keywords, risking introducing human biases and missing out on other potentially relevant aspects of the data.

Holt and Barkemeyer [16] pioneered the broadening of the geographical scope, as they considered 112 newspapers from 39 countries. This is one of the largest samples ever considered, also according to Schmidt et al. [17]. Articles were published between 1990 and 2008 and were in seven languages. For each newspaper, the monthly averages of hits on the search keywords were calculated. These allowed to quantitatively compare the coverage of sustainability and climate change over time. The analysis showed clear peaks for sustainability around the conferences of Rio de Janeiro (1992), Kyoto (COP3, 1997), and Johannesburg (2002). For both sustainability and climate change, the trend was upwards. The latter term showed a sudden upsurge after 2007. Furthermore, the authors identified several interesting local phenomena, such as the Australian coverage being up to five times larger than the global average and the US trend being downward, thus opposing the global one. Moreover, fluctuations over time were successfully interpreted by means of the *issue-attention cycles* and *punctuated equilibrium* models. This highlighted events such as the release of Al Gore’s movie *An Inconvenient Truth* in 2006 and the financial crisis starting in 2008. While these are interesting insights, the size of the dataset left the authors seemingly no choice but to use a quantitative representation of coverage. Certainly, they could relate peaks and troughs to events, but frequency alone can never reveal nuances within the text. Fewer publications on climate change can for example still imply more factual content, featuring less skepticism. This asks for a deeper analysis of the contents, yet without having to give up on data size.

In yet another quantitative study, Schmidt et al. [17] employed newspaper articles from 27 countries, including both countries with commitments to the *Kyoto Protocol* and countries that suffer strongly from the consequences of climate change. Where possible, two newspapers were incorporated from each country, to reflect different political orientations. The relevant articles from these newspapers were retrieved by using a complex search string, aggregating a wide range of terms related to climate change. In doing so, the selection was more fine-grained; articles that covered climate change without literally mentioning the term could be included while preventing noise from articles that used words like *temperature* or *heat* in another context. In the study, issue attention level, defined as the attention for climate change-related topics compared to the attention for other issues, was handled as a key metric. Apart from detecting an overall increase of climate change attention over time, Schmidt et al. [17] also found cross-national differences in the attention levels. Further analysis of these differences indicated that more vulnerable countries do not necessarily deviate from the average. Additionally, countries that have obligations under the Kyoto Protocol also have more issue attention to climate change on average. This approach yields, however, similar concerns as the study by Holt and Barkemeyer [16]: word frequency is certainly interesting, yet it cannot serve as a full representation of content just on its own. When applied correctly, this is something machine learning can overcome.

Keller et al. [14] were among the first to employ machine learning (ML), albeit unsupervised. They analyzed Indian newspaper articles for 28 topics related to climate change. The authors collected 18,224 articles from the two largest Indian newspapers, published between 1997 and 2016. In line with previous research in the field [15, 17], three keywords were used to find the articles, being *climate change*, *global warming* and *greenhouse effect*. They first assessed the proportion of articles dealing with climate change. Then, topic modeling with LDA was applied using unsupervised machine learning. The authors found that peaks in reporting on climate change strongly coincide with COP meetings (unfccc.int/process/bodies/supreme-bodies/conference-of-the-parties-cop). Overall, they revealed that reporting on climate change increased substantially between 1997 and 2016. Nonetheless, the attention in India still lagged way behind compared to other countries. Furthermore, they identified 28 climate change-related topics. *Agriculture* was the most popular at 5.02%. These topics could be structured under four overarching themes: *Climate Change and Society* (27.37%) was most popular over time, followed by *Climate Politics* (23.12%). In recent years, both became slightly less common, while *Climate Change Impacts* (17.75%) became more frequent. The less popular *Climate Change Science* (6.24%) saw rather few fluctuations. Lastly, they observed the individualization of climate change news, showing that more and more attention is drawn to individual effects and actions.

Unsupervised machine learning was also used by Chinn et al. [11]. The authors built a dataset of newspaper articles published between 1980 and 2017 in the United States, covering both national and regional newspapers. The articles were retrieved from *Lexis Nexis* by searching for the *Environment* tag and annotated using seven keyword dictionaries on different climate-related topics. The labeled dataset was then used to study the politicization of climate change news. Mentions of *Republicans* and *Democrats* were quantitatively compared to mentions of *scientists*. Their results show an increase for politicians, against a decrease for scientists. In fact, these two frequencies crossed between 2000 and 2010; every article since 2000 on average contained at least one mention of politicians, confirming the politicization trend. Additionally, the authors employed unsupervised machine learning to study polarization. First, partisan paragraphs on climate change were identified, i.e. mentioning either *Republicans* or *Democrats*. *Wordfish* (tutorials.quanteda.io/machine-learning/wordfish/) [40] was then used to determine a score for the linguistic differences between the two paragraphs. This was done for all included two-year periods corresponding to the terms of US Congress, enabling comparison over time. Results showed consistent linguistic polarization until 2011, but an increase afterward, as shown by the mentions of Republican terminology, which overtook coverage of Democratic phrases.

More recently, Hase et al. [26] conducted a novel longitudinal automated cross-country analysis of news media across the globe. They compared coverage in ten countries from the Global North and the Global South by employing unsupervised machine learning. Their study revealed both similarities and differences in coverage of climate change. Political, scientific, and (partly) societal focusing events were related to small mounts in global news media interest and countries from both areas reported on “ecological changes, climate science and the societal dimension of climate change” (p. 10). However, their study also points to important differences between countries of the Global North and the Global South, such as the first focusing more on climate science while the second paying more attention to the impact of climate change on people’s lives.

Stecula and Merkley [35] are among the few researchers who have employed supervised machine learning with support vector machines (SVMs) in the field of climate change news. They collected a dataset from four US news sources, being newspapers (1) *The New York Times*, (2) *The Wall Street Journal* and (3)*The Washington Post*, plus the news wire agency (4)*Associated Press*. 14,141 articles were collected from *Lexis Nexis* and *Factiva*. 2,177 of them were annotated for using *economic costs*, *economic benefits*, *conservative ideological* and *uncertainty* frames. Four SVM models were then trained on 80% of these, one model for each type of frame. The classification tasks were binary, either using or not using the frame. This led to accuracies around 80% across the board. The results of the SVM were then used to quantitatively analyze the share of stories that featured each frame. This showed that *economic cost* frames coincided with policy debates, such as COP meetings. There also appeared to be a downward trend for *economic cost*, against an upward trend for *economic benefits*. *The Wall Street Journal* appeared to differ from the other three sources. It featured most *economic cost* and *conservative ideological* frames. In general, *conservative ideological* and *uncertainty* frames appeared to be quite uncommon. The latter moreover turned out to be on the decline.

Despite the works of Stecula and Merkley [35], Keller et al. [14] and Chinn et al. [11], the use of ML is still rather slim in the field of climate change news. In adjacent climate change fields, there have been works that used machine learning to select scientific publications related to climate change [6, 38, 41] or to automatically categorize government policy documents, e.g. into adaptation-related classes [7, 39, 42]. Employing supervised models to uncover concrete linguistic differences between countries, political orientations or COP meetings is still unexplored. The study at hand considers that analyzing the informative features of classification and regression algorithms can be a useful contribution to the field. Doing so can help to uncover detailed differences in a large collection of climate change-related articles from a wide range of countries.

In sum, there seems to be a gap in the field of climate change news analysis, which machine learning can partly fill up. Manual analysis is currently the dominating approach, yet it suffers from some limitations: it introduces the trade-off between thoroughness and scope and comes with the risks of missing out on important data-ingrained features.

Supervised machine learning followed by informative feature analysis can help overcome these limitations. Wherever this technique has been applied so far, it was used chiefly for the prediction of artificial labels, rather than feature analysis. Classification and regression algorithms are capable of processing large amounts of data. More or less independent of the data size, by having specific training objectives, classification models can reveal data patterns that can relatively easily be analyzed.

It must be noted that the interpretation of most informative features remains a manual procedure. However, incorporating supervised machine learning into the analysis of the corpus postpones this risk until the very last phase. It also enables analysis of a larger corpus in a considerably shorter amount of time and ensures features are processed entirely systematically. This distinguishes feature analysis based on machine learning from manual analysis and makes it a fundamentally different means of studying climate change news than has been used so far. Despite the potential, machine learning is not a flawless method. It should therefore be used to complement human analysis, which is more elaborately addressed under Limitations.

The potential of analyzing models in such a way, i.e. extracting the most informative features, has already been shown in various studies, e.g. on author profiling [12] or political orientation prediction [13] for tweets. However, informative features are regularly seen as a byproduct and are presented in word clouds [13] or plots [12]. It is considered worthwhile to focus more on these informative features and perform a deeper analysis of them.

Classification models may represent more general trends than manual analyses can capture and may contain details that human analysts could potentially overlook. This can come to its full potential when systematically collected cross-country data is compared along geographical, political and temporal axes.

## Data & method

the goal of the study was to analyze differences between articles from several countries, political orientations, and COP meetings.

First, a computational data selection strategy was employed to find features inductively at scale. Subsequently, the selected data was preprocessed in order to filter out many overpredictive features.

To allow the machine learning models to be evaluated on unseen data, separate training and evaluation sets were created. As the last preparation step, features were extracted using the techniques described below.

Then, several classification models were trained and a regression model was developed to predict the COP meetings. Finally, the most informative features were analyzed.

### Data

A unique and entirely new dataset was created to analyze international newspaper articles on climate change.

The data was collected through *Nexis Uni* (internationalsales.lexisnexis.com/products/nexis-uni), the research platform from *Lexis Nexis*. Under license, this platform provides access to full-text publications from over 1,000 newspapers.

As a first step, a selection of newspapers was made. Only English-language newspapers were selected, to facilitate employment of statistical machine learning algorithms and feature interpretation by the authors. English is the most spoken language across the world and has the third most native speakers (ethnologue.com/insights/most-spoken-language/), guaranteeing sufficient coverage.

The G22 was used to narrow down the scope to sufficiently impactful countries. This group of 22 countries was mostly constituted based on financial impact (imf.org/en/About/Factsheets/A-Guide-to-Committees-Groups-and-Clubs#GS). Six countries from five continents were chosen: (1) Australia (*Australia*), (2) Canada (*North America*), (3) India (*Asia*), (4) South Africa (*Africa*), (5) the United Kingdom (*Europe*) and (6) the United States (*North America*). This was based on geographical spread, availability of English-language newspapers, and their accessibility through *Nexis Uni*.

However, since the distribution of the data featured several important irregularities (see S1 Appendix), only articles from a subset of countries (Australia, Canada, and the United Kingdom) were eventually selected for the analysis. Since these countries are all from different continents, the subset was still suitable to answer the research questions.

Each country was represented with newspapers from each end of the political spectrum (e.g. liberal/conservative) to reflect political orientation. The knowledge of the authors was used to assign these labels, substantiated by analyses from *Media Bias Fact Check* (mediabiasfactcheck.com) and *AllSides* (www.allsides.com/media-bias/media-bias-ratings) for the orientation labels. Both sources categorize newspapers using one out of five labels (left, left-center, center, right-center, right). The end result was a selection of 7 newspapers, shown in Table 1. Only newspapers classified as left-center or right-center made it to the final selection. Although the goal was to represent political opposites, the labels from the five-class scale were left intact.

**Table 1:** Overview of countries and newspapers selected for data collection and their share of articles in the dataset.

Country	Newspaper	Political orientation	Number of art.
Australia	The Australian	Right-Center	6,547
	Sydney Morning Herald	Left-Center	5,590
	The Age	Left-Center	4,321
Canada	The Globe and Mail	Right-Center	5,556
	The Toronto Star	Left-Center	4,871
United Kingdom	The Times	Right-Center	6,770
	The Guardian	Left-Center	9,917

One extra left-center newspaper was selected for Australia, considering the right-center *The Australian* has a substantially larger circulation than any left-center Australian newspaper.

As a second step, the search keywords on climate change were determined, considering that not every article that covers the subject literally mentions “*climate change*”. To generate a broader search query and prevent bias due to human-driven selection, the contents of 230 pages on *Wikipedia* (en.wikipedia.org) were retrieved and computationally analyzed using tf-idf scores to find climate change keywords. Tf-idf represents an established method for uncovering words that demonstrate relative singularity within a specified subject matter or document [43].

This involved the pages on *United Nations Climate Change conference* and *Global warming*, those for each COP meeting, and all pages linked by non-capitalized phrases on the former two pages. By defining tf-idf and document frequency thresholds, the final result was a list of 24 unigrams and 2 bigrams: (1) agreement, (2) air, (3) atmosphere, (4) carbon, (5) carbon dioxide, (6) climate, (7) climate change, (8) co2, (9) dioxide, (10) earth, (11) emissions, (12) gas, (13) global, (14) greenhouse, (15) heat, (16) ice, (17) land, (18) nations, (19) ocean, (20) protocol, (21) sea, (22) solar, (23) species, (24) surface, (25) temperature and (26) warming.

As a third step, the time frames for collection were defined, based on the occurrence of COP meetings. Keller et al. [14] have shown that news publications on climate change peaked around COP meetings. These meetings are (usually) held annually, as the main gathering of the *Conference of the Parties* (COP) from the *United Nations Framework Convention on Climate Change* (UNFCCC) (unfccc.int/process/bodies/supreme-bodies/conference-of-the-parties-cop).

The time frames around all 26 COP meetings that have taken place between 1995 and 2019 (COP 1 until 25, including the additional COP6a) were included. For each COP meeting, collection started one week before the official start and ended one week after the official ending. The average time frame covered 25 days, varying from 23 up to 27 days.

The articles were collected on a per-time frame basis. A search string was formed to retrieve full matches of one or multiple keywords, as can be seen in Fig 1 in S2 Appendix.

Unfortunately, *Nexis Uni* did not offer functionality to download more than 100 articles at once and only provided articles as formatted files (PDF). Since each time frame featured thousands of search results, collection was automatized using Python, relying on *Selenium* (selenium-python.readthedocs.io), *ChromeDriver* (chromedriver.chromium.org) and *Chromium* (chromium.org/Home). Due to hard- and software limitations, only the first 75% of articles were downloaded for the majority of COPs. With the articles already being sorted by relevance in the search results, this was not considered an issue.

The collection took place between 23 July and 4 September 2020, in reverse order of COP meetings, starting with COP25 and ending with COP1.

Next, the plain text of each article was extracted from the collected PDFs. This was achieved using Python’s *pdftotext* (github.com/jalan/pdftotext) library. The result was sanitized by removing (1) unwanted newlines, (2) multiple consecutive spaces and (3) page headings and footers.

All elements of the article were separately extracted, being (1) the newspaper (2) the date, (3) the headline, (4) the body text and (5) classifications by *Lexis Nexis*. This data was stored in 26 JSON files, one per COP meeting. Together, the full dataset for six countries contained 337,777 articles, on average 12,991 per COP meeting. 4,309 PDF articles could not be processed, 1.3% of all articles. Fig 2 in S2 Appendix shows the distribution of articles over COP meetings. Apparent is the overall increase of article counts over the years.

At this point, the dataset contained matches for at least one of the keywords. However, a single occurrence of a keyword (like “*ocean*”) does not guarantee a relation to climate change. Therefore, a procedure was used to select all articles that contained at least 3 of the selected keywords. The full procedure is explained below.

Scatter plots of absolute and unique keyword matches per COP meeting showed that keyword matches were highest for the earlier retrieved articles. Exemplar scatter plots can be seen in Figs 4, 5 in S2 Appendix.

Keller et al. [14] have shown a search strategy with multiple co-occurring keywords is useful. Hence, articles that contained less than 3 unique keywords were discarded from the dataset. While this could not guarantee matches solely covering climate change, it was considered that chances increased substantially by applying this approach, without disproportionately reducing the size of the dataset.

Filtering by 3 co-occurring keywords reduced the full dataset to 62,824 articles, on average 2,416 per COP. Fig 3 in S2 Appendix visualizes this reduction. A threshold of 4 keywords was also considered, but this was discarded as it reduced the dataset to only 31,650 articles.

Eventually, given the irregularities observed in the full dataset (S1 Appendix), only the 43,572 articles from Australia, Canada, and the United Kingdom were used for model development, shifting the average number of articles per COP to 1,676. More details are specified in Table 7 in S3 Appendix.

Three article label categories were then needed: (1) the country of the newspaper, (2) the political orientation of the newspaper, and (3) the COP meeting. The articles were already bundled per COP meeting. The former two labels could directly be derived from the publishing newspaper and assessments by *Media Bias Fact Check* and *AllSides*.

Table 2 shows the label distributions for the eventual dataset. The outstanding Australian coverage observed by Holt and Barkemeyer [16] is not seen here. Nonetheless, the overall increase over time that Schmidt et al. [17] and Keller et al. [14] described, is apparent in the dataset at hand, which is also clearly visible in Fig 3 in S2 Appendix, which visualizes the full dataset.

**Table 2:** Overview of the distributions for the country and political orientation labels.

	Country			Political orientation		
COP meeting	United Kingdom	Australia	Canada	Left-Center	Right-Center	Total
COP1	227	271	363	616	245	**861**
COP2	269	371	300	494	446	**940**
COP3	309	591	653	958	595	**1,553**
COP4	403	363	552	830	488	**1,318**
COP5	263	361	412	523	513	**1,036**
COP6	427	305	350	668	414	**1,082**
COP6a	351	468	415	667	567	**1,234**
COP7	344	467	453	710	554	**1,264**
COP8	288	360	374	546	476	**1,022**
COP9	364	437	340	640	501	**1,141**
COP10	258	392	289	535	404	**939**
COP11	558	545	442	826	719	**1,545**
COP12	673	1,087	469	1,209	1,020	**2,229**
COP13	758	1,045	615	1,380	1,038	**2,418**
COP14	575	968	391	1,037	897	**1,934**
COP15	1,136	1,531	517	1,642	1,542	**3,184**
COP16	715	814	331	1,015	845	**1,860**
COP17	718	840	402	1,087	873	**1,960**
COP18	797	663	323	942	841	**1,783**
COP19	825	710	308	1,115	728	**1,843**
COP20	981	629	350	1,283	677	**1,960**
COP21	1,695	866	507	2,080	988	**3,068**
COP22	752	522	290	827	737	**1,564**
COP23	626	549	268	759	684	**1,443**
COP24	1,107	627	352	1,036	1,050	**2,086**
COP25	1,268	676	361	1,274	1,031	**2,305**
**Average**	**642**	**633**	**401**	**950**	**726**	**1,676**
**Total**	**16,687**	**16,458**	**10,427**	**24,699**	**18,873**	**43,572**

### Preprocessing

To further optimize the data for machine learning, extensive preprocessing was employed. This entailed filtering the contents of the articles, rather than discarding articles in their entirety as in previous phases.

The preprocessing involved 8 individual filtering steps which were crafted partly during intermediate training runs of the model, as shown in S4 Appendix. Most preprocessing relied entirely on case-insensitive *regular expressions* to find and remove unwanted phrases. Named entity removal relied on *SpaCy*’s (spacy.io) tokenization, allowing to filter the data token by token. The tokens were merged into a single string again afterward.

### Splitting

To be able to evaluate the classification and regression models, not all available data was used for training. First, 20% of the data was randomly split off as *Test* set, i.e. data left unseen by all models and used for the final evaluation. The remaining 80% was further split into a *Train* set for training the models during experiments and a *Dev* set for evaluating these experimental models, again randomly and with an 80%-20% ratio. This translates as 64% and 16% of all data respectively. In the end, the combined *Train-Dev* set was used to train the final model, since the *Test* set was available for evaluation. For each split, a random seed was used to ensure randomness while keeping reproducibility. The label distributions in each subset of the data were verified to be roughly consistent and true to the distribution in the original dataset.

### Features

The main features were word and character n-grams. These were represented by tf-idf relevance scores. For each model, tf-idf scores were calculated over the whole corpus using TfidfVectorizer (scikit-learn.org/stable/modules/generated/sklearn.feature_extraction.text.TfidfVectorizer) from *Scikit Learn*. The n-gram ranges could be of different lengths, so that not only word unigrams, but also bi- and trigrams, as well as character multigrams could be included by choice during experiments. The headline and the body text of each article were handled separately, allowing the use of different settings for each element.

### Classification

#### Architecture

Classification was implemented using *Scikit Learn*’s LinearSVC (scikit-learn.org/stable/modules/generated/sklearn.svm.LinearSVC) class. This features a linear implementation of support vector machines (scikit-learn.org/stable/modules/svm) (SVM), which is a popular and effective architecture in natural language processing. The default settings for LinearSVC were used so that the focus could be on feature generation.

For each of the three labels (countries, political orientations and COP meetings) an independent classification model was trained. All three models were trained consecutively during experiments with different features and settings. This allowed to constantly evaluate the impact of changes across aspects.

During experiments, the models were trained in a single run on the *Train* data and consecutively evaluated on the *Dev* set. Eventually, a final model was trained using the optimal configuration on the *Train-Dev* data and evaluated on the so far unseen *Test* set. Performance of each created model was evaluated by means of Precision, Recall and F1-score.

The goal was to determine and analyze the most informative features, i.e. the textual features that contribute most to the model’s decision (as done before, for example, by [12, 13]). This is considered an objective way to determine which terms are characteristic or rather uncharacteristic for each country, political orientation and COP meeting. In a broader sense, this allows to uncover topical and terminological differences between the articles for these labels. While this analysis is purely textual and does not consider context or meaning, it can still provide insight into a large dataset in a neutral manner.

Concretely, in order to assess the contribution of each single feature, the model’s *coefficients* were extracted. In Scikit Learn, coefficents can be easily accessed using LinearSVC.coef_. A more detailed explanation can be found on scikit-learn.org/stable/modules/svm.html. Both positive and negative *coefficients* are generated for each class. These are the weights of the features, with absolutely higher weights indicating a stronger contribution of the corresponding feature to the models’ decision.

Since a lot of features were generated, only the ones with the most positive and negative *coefficients* were presented. For each given class (for example the country “Australia” in country detection), features with positive weights can be seen as strongly characteristic for that class, and ones with negative weights as uncharacteristic. This allowed to analyze the textual characteristics of each class.

To provide more perspective for the scores that would be yielded by the SVM, a baseline was first generated using the DummyClassifier (scikit-learn.org/stable/modules/generated/sklearn.dummy.DummyClassifier) class. A stratified strategy was employed, which leads to random predictions solely based on the class distribution within the training data. This can be interpreted as educated guessing. The SVM models should thus at least outperform this baseline to be considered useful.

When trained on the *Train-Dev* set and evaluated on the *Test* data, the baseline yields a macro F1-score of 0.33 for countries, 0.51 for political orientations and 0.03 for COP meetings. These scores are as expected for random guessing with 3, 2 and 26 classes respectively.

#### Experiments

Several experiments were conducted to determine the most useful combination of features and preprocessing steps. This assessment was based both on performance scores and most informative features. The majority of experiments were executed on the *Peregrine* HPC cluster of the University of Groningen, and some additional runs were performed on *Hábrók*, its successor (rug.nl/society-business/centre-for-information-technology/research/services/hpc/facilities/peregrine-hpc-cluster). The accuracy and macro F1-scores yielded by all 18 experiments can be seen in Table 8 in S5 Appendix. These models were trained on *Train* data and evaluated on the *Dev* set.

For some final experiments, word embeddings were incorporated into the feature set. While this type of feature is not useful in answering the research questions at hand, embeddings have proven to be powerful predictors in text classification [44]. Therefore, the impact of implementing them (or conversely, avoiding their use) was evaluated. The 300-dimensional word embeddings vectors from Google’s word2vec project (code.google.com/archive/p/word2vec/) were used here as averaged document vectors. Performance improved only slightly or not at all when these vectors were added to the feature set, which justified the use of solely textual features for further model development, given that explainability was favored over performance in this context.

Taking all experiments into account, the configuration with word 1–3-grams and the full preprocessing strategy (Experiment 15) was chosen as the final configuration for all three aspects. Tables 3–5 show the classification reports for the final model. This was trained using this optimal configuration on *Train-Dev* data, and then evaluated on the *Test* set. This led to scores that were only slightly deviant from those in the experimental phase, where training was performed on *Train* and evaluation on *Test* data.

**Table 3:** Classification report of the final model for country labels.

	precision	recall	f1-score
**Australia**	0.89	0.89	0.89
**Canada**	0.94	0.88	0.91
**United Kingdom**	0.88	0.92	0.90
**Macro average**	0.90	0.89	0.90

**Table 4:** Classification report of the final model for political orientation labels.

	precision	recall	f1-score
**left-center**	0.79	0.90	0.84
**right-center**	0.84	0.68	0.75
**Macro average**	0.81	0.79	0.80

**Table 5:** Classification report of the final model for COP meeting labels.

	precision	recall	f1-score
COP1	0.58	0.37	0.45
COP2	0.46	0.30	0.36
COP3	0.41	0.52	0.46
COP4	0.51	0.49	0.50
COP5	0.34	0.24	0.28
COP6	0.38	0.23	0.28
COP6a	0.41	0.35	0.38
COP7	0.41	0.39	0.40
COP8	0.56	0.23	0.32
COP9	0.25	0.22	0.23
COP10	0.33	0.13	0.18
COP11	0.43	0.27	0.33
COP12	0.39	0.50	0.44
COP13	0.42	0.49	0.45
COP14	0.46	0.47	0.47
COP15	0.50	0.66	0.57
COP16	0.51	0.34	0.41
COP17	0.42	0.43	0.42
COP18	0.56	0.41	0.47
COP19	0.48	0.49	0.49
COP20	0.54	0.49	0.51
COP21	0.46	0.76	0.58
COP22	0.53	0.49	0.51
COP23	0.63	0.42	0.50
COP24	0.59	0.59	0.59
COP25	0.53	0.74	0.62
**Macro avg**	0.46	0.42	0.43

### Regression

#### Architecture

Apart from classification, an implementation of regression was developed for the COP meeting labels. These labels are numeric and have a temporal nature. Thus, it is better to predict a meeting closer to the real label than one that is several years off. This is not taken into account when using classification with F1-scores for evaluation, where a prediction can be either wrong or right. A regression model is therefore considered a more suitable way to predict the COP label. This type of model leads to a continuous number and uses the difference from the correct value for evaluation, called the error.

Regression was implemented using the LinearRegression (scikit-learn.org/stable/modules/generated/sklearn.linear_model.LinearRegression) class from *Scikit Learn*. This was integrated in the existing pipeline and the approach to preprocessing, splitting the data and generating features remained the same consequently.

The COP meeting labels were converted to numbers. COP6a was represented as 6.5 and all other meetings as their round edition number. The models were then trained using similar n-gram feature combinations as in the classification experiments.

The performance of the resulting models was evaluated using the *Mean Absolute Error* (MAE), *Mean Squared Error* (MSE) and *Root Mean Squared Error* (RMSE).

Like for classification, the most informative features were extracted by linking the model’s coefficients to the original features. For their nature to predict continuous numbers, regression models only yield overall coefficients. These are less insightful than the per-class coefficients from classification. However, the coefficients do indicate what drew the model towards a higher or a lower prediction.

Unlike the classification model, no baseline was defined for regression. A rule-based regressor like DummyRegressor (scikit-learn.org/stable/modules/generated/sklearn.dummy.DummyRegressor) relies on always predicting a single value, such as the mean or the median of the labels. Having as many as 26 COP meetings that moreover have a temporal nature, such a model would inevitably produce high errors that are effectively meaningless.

#### Experiments

To determine an optimal configuration for the regression algorithm, a couple of experiments were conducted. Since findings similar to those in the classification experiments were expected, the intermediate pre-processing strategies were not tested with the regression algorithm. The performance of the models was compared by use of all three error metrics, being MAE, MSE and RMSE. The error scores and R^2^ for the final model using the optimal configuration are in Table 6. Table 9 in S5 Appendix shows the scores from all experiments.

**Table 6:** Error scores of the final regression model for COP meeting numbers.

	MAE	MSE	RMSE	R^2^
**COP meeting**	2.95	16.59	4.07	0.65

### Reproducibility

All code employed, plots generated and most informative features yielded in this study are available on DataverseNL: doi.org/10.34894/AMISPR. The dataset was collected from *Nexis Uni* under license of the University of Groningen, and is therefore not publicly available. The data can be reproduced by licensees using the provided scripts.

## Results

The most informative features yielded by the three classification models (COPs, countries and political orientations) and the regression model for COPs were analyzed to uncover what each model had learned from the data.

The most positive and negative features for each class were listed, with the extremest coefficients first. Herein, the positive features can be seen as characteristic of that class, and the negative ones as uncharacteristic.

For the COP classification model, this was limited to 50 features from each side of the spectrum. A limit of 100 was applied for the political orientation and country models. This means that 2,600, 600, and 200 most informative features were extracted from the three models respectively.

The 250 most positive and negative informative features from the COP regression model were extracted, i.e. 500 in total. Seeing that a regression model only yields a single set of coefficients, inherently not structured by class, it was useful to extract more features.

These features were manually analyzed for topical and terminological differences. This was largely a qualitative analysis of the n-grams, subject to the scope of expertise and knowledge of the study’s authors. Some quantitative aspects were considered in this analysis as well, such as the height of the corresponding coefficient and the overlap of features across classes. For regression, coefficient height played a larger role, as to comprehend the single set of features yielded.

The analysis below provides an exploration of the features aiming to answer the research questions and assess the potential of the methodology, including finding room for future improvements.

Apart from the analysis, the full lists of most informative features must in itself be seen as an important result of this study. These can be accessed through OSF as found under Reproducibility. Extended details about the performance of the classification and regression models can be found under Method in S5 Appendix.

### Countries

The model to distinguish countries scored an overall F1-score of 0.90. This was considered a trustworthy performance, thus feature analysis could proceed.

Among these features, some keywords covered climate change directly. These are useful in answering the tertiary research question on countries (“*What major topical and terminological differences can be found between countries and how could they be explained?*”).

For structure, four categories were defined, being the mere terminology to describe climate change, its impact, the causes and taken measures. It seems likely that these differences are meaningful and interesting.

- **Terminology**
  Climate change may be a global problem, but it is not necessarily a global term. It turns out that the three countries use different terms to refer to the concept. This is in line with the findings from Grundmann and Scott [15]. Whereas *climate* is a characteristic term for the UK, *greenhouse* is more common in Australia. This can be augmented as *greenhouse emissions*, while this seems to be *carbon emissions* in the UK. In Canada, *environmental* is used, which is less specific.
  **UK:** climate, carbon emissions
  **CA:** environmental
  **AU:** greenhouse, greenhouse emissions
- **Effects**
  The countries also describe different words that refer to the effects of climate change. While Australia seems to be worried about *bushfires* and *drought*, the UK takes on *deforestation* and *wildlife*. It appears Australia also considers longer-term effects such as *asylum seekers* and a *global financial crisis*. At the same time, *vacation* could suggest what the Canadians fear.
  **UK:** deforestation, wildlife
  **CA:** vacation
  **AU:** bushfires, asylum, asylum seekers, global financial crisis, drought
- **Causes**
  Various causes of climate change also came up. Australia speaks about *mining*, while Canada mentions *oil sands* and *natural gas*. The UK brings up *flights*. Canada also seems to link *globalization* to the topic. Australia is recognized by the absence of *snow* and *oil*, which therefore seem characteristic for the other countries.
  **UK:** flights
  **CA:** oil sands, natural gas, globalization, pipeline
  **AU:** mining
  **UK/CA:** oil, snow
- **Measures**
  Lastly, there are different measures against climate change that characterize the countries. The UK speaks about *low carbon*, apparently leaving the means by which that must be achieved in the middle. Australia mentions *uranium*, a carbon-free form of energy production. This is likely related to Australia having the world’s largest resource of uranium, and being one of the largest exporters of uranium worldwide (world-nuclear.org/information-library/country-profiles/countries-a-f/australia.aspx). This shows that characteristics of countries carry over into their national news coverage. The concept of a *carbon tax* is uncharacteristic for the UK, hence it is apparently more of a discussion item in Canada and Australia.
  **UK:** low carbon
  **AU:** uranium
  **CA/AU:** carbon tax

Apart from these climate change-related features, the majority of most informative features were seemingly unrelated to climate change. Yet, they were used by the model to distinguish countries. Only a handful of features were analyzed (UK: 7, CA: 6, AU: 11), while 200 most informative features were extracted (100 positive and 100 negative) per country. Percentually, that means only 3% to 6% of features were found directly relevant to climate change. Most of these non-climate change features can be described as ortographical differences (e.g. *program* vs. *programme*), lexical variations (e.g. *transport* vs. *transportation*), local currencies (e.g. *pounds* vs. *dollar*), political terminology (e.g. *treasurer* vs. *chancellor*), national sports (e.g. *football* vs. *soccer*), and geographical and demographical terminology (e.g. *province* vs. *state*). No feasible explanation could be found for plenty of other features. The full list of extracted features is accessible as instructed under Reproducibility.

In sum, our results, in line with prior research in the field, point to important differences among the countries with regard to the effects, causes, and solutions to climate change. For example, while for Australia “*mining*” is shown to be a distinctive cause of climate change, in the case of UK this is “*flights*” and for Canada “*oil sands*” comes up. These features seem to respond to specific economic and political contexts [34] of the countries: London serves as the largest aviation hub in the world by passenger traffic (en.wikipedia.org/wiki/List_of_busiest_airports_in_the_United_Kingdom), Australia is the world’s largest producer of iron ore and bauxite and the second largest of gold (en.wikipedia.org/wiki/Mining_in_Australia), and Alberta’s (Canada) oils sands, is the world’s third-largest proven oil reserve at 170 billion barrels (nationalgeographic.com/environment/article/alberta-canadas-tar-sands-is-growing-but-indigenous-people-fight-back).

### Political orientations

For the political orientations model, the average F1-score was 0.80. This was somewhat lower than for the countries but still meant the vast majority of articles could correctly be classified. Hence, analysis of the most informative features was also sensible for this model.

It showed the same picture as for the countries: the model relies on many features that indicate differences irrelevant to climate change. Moreover, the model also relies on features that were earlier found to be indicative of individual countries. This is surprising, for every country is represented on both sides of the political spectrum. However, some interesting observations can be made both literally and figuratively between the lines of these less relevant features. These observations can help answer the second research question (“*What major topical and terminological differences can be found between political orientations and how could they be explained?*”).

When it comes to climate change-related features, the right and left show several contrasts. In general, it turns out easier to find related terminology that is indicative of left-oriented newspapers. This could suggest that these also cover the subject more frequently.

It also raises the question of what the model picked up instead. The share of features that seem to be country indicators or political themes is especially larger for right-oriented articles. Apart from those phrases, the features seem highly irrelevant.

The relevant keywords can be characterized using a similar structure as for the model for countries:

- **Terminology**
  The right and the left side of politics seem to use different words for the matter of climate change. The right just seems to name it by *the climate change*. The left is more outspoken, by referring to a *crisis*. Furthermore, they use *greenhouse* and *environment* more. Seeing that features that were indicative of countries came back in this model, it could however be that these insights were also subject to this effect.
  **L:** greenhouse, environment, crisis
  **R:** the climate change
- **Causes**
  The model also picked up on the causes of climate change. The left refers to *polluters*, who might in their perspective have to pay. This relates to the phrases *transport* and *fossil fuel*. The right side only seems to consider *aircraft* a worthy cause of the changing climate.
  **L:** polluters, transport, fossil fuel
  **R:** aircraft
- **Measures**
  When it comes to measures against climate change, the right is again overtaken by the left. Only *carbon tax* was found for right-oriented articles. This is rather surprising, but could well mean that the right articles argue against it. Liberal journalism speaks about *energy efficiency*, *climate talks* and *cooperation*, like during COPs. Lastly, *sustainable* initiatives also seem to be on their agenda.
  **L:** energy efficiency, climate talks, cooperation, sustainable
  **R:** carbon tax
- **Effects**
  The model did not value many features related to the effects of climate change. Only *forests* as found indicative for left-oriented newspapers.
  **L:** forests

Again, apart from the features analyzed (L: 11, R: 3), the majority of the 100 most informative features extracted for each orientation were unrelated to climate change. The share of relevant features is 3% for right-oriented newspapers and 11% for left-oriented ones. Several non-climate related features that were earlier observed as informative for countries, were also informative for the political orientations. Examples are local currencies, lexical variations and political differences. Moreover, specific political themes come up, that are typically favored by specific sides of the political spectrum (e.g. L: *housing*, *schools*; R: *business*, *market*). Please refer to Reproducibility, for access to the full list of extracted features.

In a nutshell, our findings reveal a clear unbalance between the features of left-oriented newspapers (11) and those of the right-oriented newspapers (4). Furthermore, our results point to different causes (e.g. L: *fossil fuel*; R: *aircraft*) and measures (e.g. L: *energy efficiency*; R: *carbon tax*) between the newspapers. Thus, in line with findings by Chinn et al. [11] and Potthast et al. [10], our research also reveals differences due to political orientations.

### COP meetings

The model for COP meetings scored a much lower macro average F1-score of 0.43. This inevitably means that the model has learned features that were not effective in distinguishing the labels in the *Test* data.

The average F1-score comes about from a variety of F1-scores per label. Some meetings could hardly be detected (COP10, 0.18), while others still scored a fair performance (COP25, 0.62). It was considered that all labels scoring below 0.50 on F1 were too unreliable to be analyzed. Only 8 labels scored above this threshold, being COP4, 15, and 20 until 25. This list was augmented by COP3 (F1 0.46), since the crucial Kyoto Protocol (unfccc.int/kyoto_protocol) was signed during this edition.

It is remarkable that when compared to the models for countries and political orientations, a greater share of the most informative features yielded by the COP model seems to be related to climate change. This contributes to answering the research question for this aspect (“*What major topical and terminological differences can be found between COP meetings and how could they be explained?*”). Some COP meetings stand out with many features related to climate change, while others fall short and show many other topics. It can well be that this is caused by the varying relevance of the meetings. Some meetings seemed to have little effect, while others were quite impactful, like COP15 due to *Climategate* (factcheck.org/2009/12/climategate/) and COP21 for the Paris Agreement (unfccc.int/process-and-meetings/the-paris-agreement/the-paris-agreement).

There seem to be some clear differences between the early and more recent COP editions. Among other things, this seems to concentrate around the terminology used to refer to climate change—especially when it comes to urgency –, the attempts made to reduce the effect, and the output of the conferences itself.

Examples are provided in terms of positive (in ${\text{green}}^{+}$) and negative features (in ${red}^{-}$) for the COP meetings that featured relevant phrases. Positive features are selected by the model as characteristic for that class, while the negative ones have been identified as uncharacteristic. COP meetings are referred to by numbers in these examples.

- **Terminology**
  When it comes to the terminology used to describe climate change, a threefold shift seems to have occurred.
  During COP3 in 1997, the term *climate change* was not indicative yet. This suggests that there were concerns, though no trend of change was being described yet. Instead, merely the *emissions* of *carbon dioxide* as well as the resulting *greenhouse* effect and *global warming* seem to have been the subjects of discussion, judging by the most informative features.
  This changed later, seeing that the term *climate change* was positively informative for COP15 in 2009. The terms *global warming* and *greenhouse* were no longer highly indicative for subsequent COP meetings 20 and 22 respectively.
  For COP25 (2019), yet a new level of terminology was observed. Here, the term *climate change* was no longer highly indicative. To the contrary, the terms *climate crisis* and *climate emergency* were. Similarly, *global warming* was no longer observed in the most informative features, but the more intense *global heating* was. It seems the urgency of the matter started to sink in at this point.

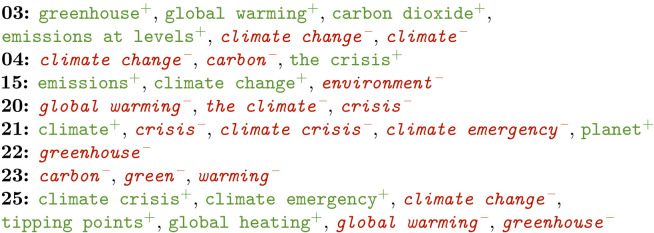
- **Conferences**
  Related to conferences, several features stand out as well. During the earlier meetings COP3 and 15, *summit* was widely used and so was *treaty*. Later on, these words became negative indicators for COP22 and 23 respectively and were thus apparently not used widely anymore. It can well be that the term *treaty* had been replaced by *climate deal* and *climate agreement* at the time of COP21 and 22, since the output of COP21 was not a treaty but rather the Paris Agreement. COP21 also shows *goal* as informative, which likely refers to the target to limit global temperature rise to 2°C.
  The meeting of COP15 was a special one, due to the occurrence of Climategate just ahead of its start. More than 1,000 stolen emails were leaked in an attempt to argue that scientists were manipulating their studies to falsely prove global warming (factcheck.org/2009/12/climategate/). The media clearly picked up on this, judging by the occurrence of *leaked*, *sceptics* and *deniers* in the most informative features.
  During the same edition, the concept of *emissions trading* became indicative. This related to the Emissions Trading System of the EU (ec.europa.eu/clima/policies/ets_en) that strives to lower European carbon emissions by the principle of *cap and trade*.
  A comparable concept is that of *carryover credits*. These would entitle countries to excess carbon emissions since they previously reduced emissions further than the Kyoto Protocol required. This is especially a topic in Australia, which attempts to keep these credits valid under the new regulations of the Paris Agreement (ec.europa.eu/clima/policies/ets/credits_en).

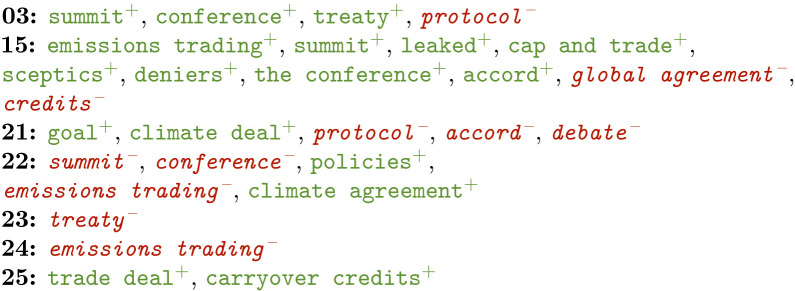
- **Effects**
  Various effects of climate change were also found indicative of COP meetings. No real trend could be seen, apart from what might be an overall terminological change from *fires* towards *bushfires*. What is striking, is that COP25 mentions by far the most effects. This is in line with the earlier suggestion that urgency got more traction during this edition.
  Several terms can, to a certain extent, also be explained by natural events occurring during a given COP edition. For example, there were particularly severe *bushfires* in Australia (disasterphilanthropy.org/disasters/2019-australian-wildfires/) and a volcanic *eruption* on White Island in New Zealand (britannica.com/event/White-Island-volcanic-eruption-of-2019) during COP25 in 2019.

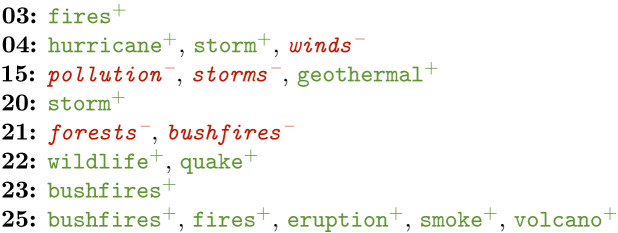
- **Causes**
  Where there are effects, there are causes. Logically, several of these were found within the features for the COP meetings. Again, it was hard to notice a blatant trend in these. The explosion for COP25 was not observed either. Rather, more features were found for intermediate meetings like COP20 and 21. It may be that causes were not specifically addressed before and are more left to common sense by now. In extension, the attention may increasingly be drawn towards measures against climate change.

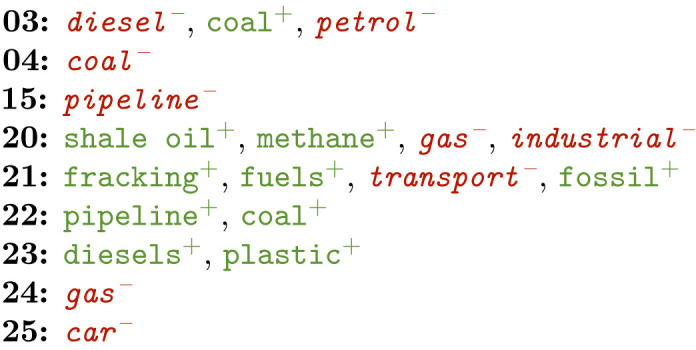
- **Measures**
  Looking at measures, none were found for earlier COP meetings. This could support the idea that the attention is shifting from observing causes to acting with measures. COP21 clearly shows *solar* and *clean energy* as indicative. COP23 features *energy storage*. By COP25, *clean energy* had become a negative feature, thus being uncharacteristic for the class. Instead, *transition*, *electric* and *emissions reduction* had become positively indicative, i.e. characteristic for articles published around COP25.

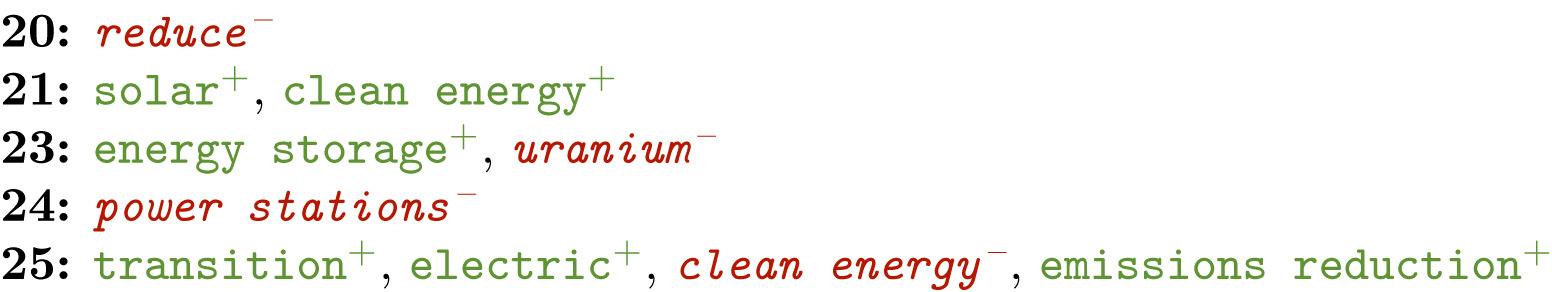

Only 3% to 19% of the extracted most informative features were deemed relevant to climate change and have thus been analyzed (**3:** 14, **4:** 7, **15:** 17, **20:** 14, **21:** 18, **22:** 10, **23:** 14, **24:** 3, **25:** 19). While most features cannot be plausibly explained, several can be related to historical events co-occuring with COP meetings. Examples are the Ottawa Treaty (un.org/disarmament/anti-personnel-landmines-convention/) on land mines (COP3), the Ebola pandemic in Western Africa (COP20), the advance of Islamic State in Iraq and Syria (COP21), the presidential election of Donald Trump (COP22), the mass displacement of the Rohingya (COP23), the Brexit agreement (COP24), and the impeachment of Trump (COP25). All features can be accessed, as indicated under Reproducibility.

Apart from a classification model, the COP meetings were also predicted using regression. While the classification model had yielded rather low scores, the regression model seemed to be performing quite well.

This results from the fundamentally different approach of the two algorithms and the related means of evaluation. Whereas a prediction of a class can only be wrong or right, a numeric prediction by regression is evaluated with its precise error from the gold standard. Interpreting the MAE of 2.95, one can say that the regression model is on average roughly 3 COP editions off. RMSE corrects for larger variance and is 4.07 for this model. This can roughly be explained as the model being 4 editions off, although the metric is in fact more complex. Considering that there are 26 COP meetings, not every edition is as impactful and most characteristics change gradually rather than at once, these scores shall be considered acceptable.

The most informative features of regression models are only based on a single set of positive and negative coefficients. Since n-grams are represented by positive *tf-idf* importance scores, these respectively refer to an increase or a decrease of the predicted COP meeting number. Looking at the top of the positive and negative features, conclusions can therefore only be drawn in terms of phrases for later and earlier COP meetings. This seems to reflect many of the findings done with the classification model. Several examples are given below, for earlier (negative features, in ${red}^{-}$) and later COPs (positive features, in ${\text{green}}^{+}$).

- **Terminology**
  The terminology found using classification comes back in the regression model. Terminology has shifted to become more descriptive of a trend (*greenhouse* vs. *climate*) and more extreme (*climate crisis*). It also seems that *carbon dioxide* is now more frequently referred to merely as *carbon*.
  **Earlier:** greenhouse, global warming, greenhouse emissions, carbon dioxide, environmentalists, earth, climatic, environment, greenhouse effect, carbon dioxide emissions
  **Later:** climate, planet, carbon, climate crisis, emissions, CO2, footprint, climate emergency, carbon emissions
- **Causes**
  Similarly, phrases that describe the causes of climate change largely matched those found before. It does seem that describing causes is something of the later editions. However, this is in fact sound with the findings that intermediate meetings like COP20 until 23 are characterized more in terms of causes.
  **Earlier:** petroleum, gas, traffic
  **Later:** aircraft, shale, fracking, diesel, air pollution, plastic, coal, methane, drilling, vehicles, shale oil, oilsands
- **Effects**
  Some effects were found in the most informative features, though much less than for the classification model. An interesting word that is yielded, is *ozone*, referring to the ozone layer hole. This was more of a concern earlier but seems to be on its return under the effect of the Montreal Protocol (unep.org/ozonaction/who-we-are/about-montreal-protocol). It is shown that the media wrote a lot more about it during earlier COP meetings.
  **Earlier:** ozone, smog, air conditioning
  **Later:** bushfires
- **Measures**
  The potential trend that measures are becoming more and more subject of attention seems to be reflected by the yields of the regression model. No features that describe measures were found on the negative side of the coefficient spectrum, whereas quite some could be detected with positive scores. These phrases are predominantly the same as for classification. Though *sustainability* and *electric vehicles* are interesting additions.
  **Later:** electric, electric vehicles, battery, and solar, renewables, sustainability, emissions reduction

Like the classification models, the regression model mostly produced features unrelated to climate change. These are much like those yielded in classification, but were not analyzed in-depth. Of the 250 positive and negative features extracted, 6 to 12% were analyzed as relevant to climate change (Earlier: 16, Later: 29). The full list of features is available, as described under Reproducibility.

## Discussion & conclusions

This study has uncovered relevant topical and terminological differences between newspaper articles on climate change in Australia, Canada, and the United Kingdom. In doing so, it has made use of supervised machine learning. Since most of the studies on climate change news focus on single countries and make use of manual content analysis [17], we believe that our research can positively contribute to the development of the field.

The study aimed to find a response to three research questions: Are there major topical and terminological differences between the articles on climate change by newspapers from different countries (RQ1)?; Are there major topical and terminological differences between the articles on climate change by newspapers with different political orientations (RQ2)?; Are there major topical and terminological differences between the newspaper articles on climate change during different COP meetings (RQ3)?

The results of our machine learning model reveal that countries can be automatically classified accurately, with a macro F1-score of 0.90. Based on these findings and the analysis of most informative features, it can also be concluded that there are topical and terminological differences between these countries (RQ1). These differences have to do with climate change terminology, and its effects, causes, and measures.

The model for political orientations had the second best fit, scoring a macro F1-score of 0.80. This result allows us to conclude that it is possible to automatically classify articles into the political orientation of their newspaper. This means that distinctive features (topical and terminological) for political orientation also exist in the corpus (RQ2). These differences, in line with the findings reached in the country model, are terminological and have to do with the causes, measures, and effects of climate change news articles.

The results of the models for differentiating between COP meetings are slightly less outspoken. It is not possible to convincingly distinguish every COP edition. Indeed, the macro F1 score was 0.43, although it greatly differed between the meetings. This signals that not every meeting was equally relevant. However, it can be concluded that the model detected certain differences, seeing that the baseline was outperformed.

When assessed as a regression task, taking into account the temporal nature of these labels, an RMSE of 4.07 was yielded. Thus, the COP meeting can properly be estimated up to roughly half a decade. Regression shows to be a more suitable method to predict the COP meeting for newspaper articles. This leads us to conclude that it is possible to automatically predict the COP meeting sufficiently accurately.

Some COP meetings do indeed show relevant topical and terminological differences, while others hardly do (RQ3). Those that do feature these differences show a clear terminology transition over the years and differences in conference-related phrases, as well as effects, causes, and measures. It also becomes clear that later COPs show more relevant features and more urgent phrases than earlier ones. Specifically, terminology has shifted to become more descriptive of a trend (*greenhouse* vs. *climate*) and more extreme (*climate crisis*); describing causes is something of the later editions (e.g., *fracking*, *drilling*, *oilsands*); and measures are becoming more and more subject of attention (e.g., *electric vehicles*, *renewables*, *emissions reductions*). The latter is in line with findings by Keller et al. that climate change news is individualizing, with a growing focus on individual actions [14].

Apart from features, this study has yielded a new dataset that can be analyzed in many ways to uncover new insights concerning newspapers’ approaches to climate change. The data also lends itself for e.g. headline generation or manual qualitative analyses. Future research could for example make use of neural networks [45] to outperform our current models or employ word embeddings [44] to predict the political orientation of the newspapers. On the other hand, studying the data through the use of manual discourse analysis will help unfold different insights that automated methods struggle to detect, e.g. some contextual characteristics of the corpus.

All in all, the research constitutes a first step in exploring the automatic detection of differences in news articles with regard to countries, political orientations, and COP meetings. The study contributes to the use of supervised machine learning for highlighting discriminative features on how newspaper articles approach climate change globally.

Future works should pick up where this study left off. Above all, we consider it essential to further bridge our results to a wide array of previous works, and see plenty of opportunities to do so. For example, studies focusing on scientific publications [6, 38, 41], government policies [7, 39, 42] or news articles [11, 14–17, 26, 30, 31, 35]. It will be insightful to put the features yielded in the study at hand further into context and to see whether and how results align with earlier findings.

Furthermore, future works should expand our analyses to other countries and news articles from the most recent COP meetings (26, 27 and 28). Now that the feasibility of the approach has been proven, we also consider it worthwhile to focus on further finetuning machine learning model development in ways we did not cover, e.g. by tuning the SVM’s hyperparameters (scikit-learn.org/stable/modules/generated/sklearn.svm.SVC.html) and using cross-validation (scikit-learn.org/stable/modules/cross_validation.html). The creation of a protocol for feature analysis should also be taken into account, to further structure annotation of the yielded most informative features. Another opportunity lies in more directly comparing feature overlap from our classification and regression models for COP meetings.

## Limitations

While our exploratory research has yielded interesting insights into how news media are approaching climate change across countries, we also acknowledge several limitations that accompany our investigation.

First, we consider machine learning a way to reduce the chance of introducing human bias in the analysis of newspaper articles and have presented the technique as such. Our methodology proves that it is indeed possible to systematically find, collect, and digest articles using classification and regression models. Nonetheless, the analysis of these features was a manual process, based on our own general knowledge and common sense. We are convinced that more systematic approaches to analyzing the meaning and context of features yielded by our models can be designed. For example, we strongly suggest bridging our results with findings by preceding publications on climate change news [11, 14–17, 26, 30, 31, 35], scientific publications [6, 38, 41] or government policies [7, 39, 42]. Also, we stress the need to pair the features we present with the knowledge that experts in the field of climate change have.

It must also be pointed out that the machine learning methodology presented in this study does not take into account context beyond the features themselves. Word order is only preserved within multigram features. Beyond that, it is impossible to determine from the model itself how certain most informative features are used in the original text and substantiate why they appear as significant for a given class. It may be that a feature that is significant to the model is not so apparent to a human reader, e.g., due to its context or frequency. We believe this can also be overcome by further analysis, be it using a deeper analysis of the dataset we created, or using a broader comparison with other findings.

We see analysis of most informative features yielded by machine learning models as a useful addition to the field of climate change. We would like to encourage researchers in this field to incorporate the techniques we presented into their own qualitative studies. This could improve reliability and uncover patterns that may otherwise remain unseen by the human eye. We do not mean to suggest that manual analysis can be entirely replaced by machine learning. Rather, we believe the two can complement each other. A bottom-up approach such as the one we have presented, in which the content is leading, can prompt researchers to start looking for differences they might overlook in top-down analyses.

Second, a more technical limitation of this proof-of-concept approach is the lack of parameter tuning for both the classification and regression models, e.g. using an RBF instead of a linear kernel, or optimizing C for the support vector machines classifier. We have focused on collecting the data and optimizing the preprocessing of features, but have deliberately chosen not to involve the parameters of the employed LinearSVC and LinearRegression estimators. It may well be that parameter tuning can further enhance the performance of the models and can thus lead to more representative features. We therefore highly recommend incorporating even the mildest parameter tuning into future work.

We also believe that the preprocessing strategy designed in this study can further be improved upon. For example, features that differ e.g. orthographically, lexically and geographically, and are currently used by the model to distinguish countries should be filtered out more effectively. Named entity recognition also leaves room for improvement, which can possibly be achieved by using more recent, larger (transformer-based), and news-related models from *SpaCy*.

Lastly, our systematic data collection approach was based on 3 co-occurring keywords extracted from *Wikipedia* articles, to raise relevancy towards climate change. It can however not be guaranteed that all collected articles explicitly cover the topic. Ambiguity of keywords and tricky keyword combinations may have captured articles on other subjects as well. We deliberately chose not to further reduce the size of the dataset by filtering for 4 co-occurring keywords, aiming to balance accuracy and data size. It was considered that a larger amount of data could mostly cancel out noise from less relevant articles.

Despite the limitations that this study faced, we do consider it a successful proof of concept for feature analysis based on machine learning models in the field of climate change. We hope that future work will overcome several of these limitations, but moreover that machine learning will start to become a useful tool for researchers in analyzing textual patterns in climate change news and related documents.

## Supporting information

S1 Appendix Country selection. Detailed explanation of the country selection process.
(PDF)

S2 Appendix Keyword selection. Figures detailing the keyword selection process.
(PDF)

S3 Appendix Data description. Table of further statistics describing the data.
(PDF)

S4 Appendix Feature selection. Further details on the feature selection process.
(PDF)

S5 Appendix Experiments & performance. Tables detailing model experiments and the resulting performance.
(PDF)
