# Supplementary material for: Learning from climate change news: Is the world on the same page?
Source: PLoS One. 2024 Mar 20;19(3):e0297644. doi: 10.1371/journal.pone.0297644 (PMC10954114; doi:10.1371/journal.pone.0297644)
Supplement: S1 Appendix — Detailed explanation of the country selection process. (PDF) [file pone.0297644.s001.pdf]

Appendix 1: Country selection

Explanation of irregularities encountered in the full dataset, leading to the discarding of data for three countries.

The full collected dataset contained data for six countries (Australia, Canada, India, South Africa, the United Kingdom and the United States). After collection and annotation, several irregularities were observed in the data distribution. Eventually, these were reason to use only a subset of the countries for model development (Australia, Canada, the United Kingdom).

Inspection of the data made clear that older publications in newspapers from both India and South Africa were unavailable on *Nexis Uni*. This led to an evident deficit in the total number of collected articles from these countries. The first retrieved publications from South Africa are from 2008, the first Indian article is from 2010. The majority (90%) of collected Indian articles was published in *The Times of India*. Furthermore, the overall imbalance between left-center (65%) and right-center (35%) oriented articles was apparent.

First, the data for South Africa and India was set aside, since their significant underrepresentation would be too detrimental for the machine learning models. This would either lead to unfair comparison, or the number of articles for the other four countries would have to be reduced to match that of India (3,361) or even South Africa (506). That would in turn leave too small samples for the model to analyze. Additionally, earlier years would have to be ignored across the board to prevent biased models, since the deficit was partly caused by a lack of data from this period.

Second, articles from the United States were not considered in the models, since it was represented by left-center newspapers only. This would inevitably introduce a bias into the models: Clearly into that for political orientation, but possibly more latently into the other models as well.

All of the above does not mean that the collected data from the Unites States, India and South Africa is not useful. The full annotated dataset is still considered a valuable source that can facilitate various other tasks. However, the research questions at hand, use of a subset was considered the only valid approach. When compared to Table 2, it is clear that the use of this subset managed to repair the extremeness of these irregularities, also with respect to political orientation.
