# Supplementary material for: Learning from climate change news: Is the world on the same page?
Source: PLoS One. 2024 Mar 20;19(3):e0297644. doi: 10.1371/journal.pone.0297644 (PMC10954114; doi:10.1371/journal.pone.0297644)

Appendix 2: Keyword selection

Fig 1. *Nexis Uni* search string representing the 26 selected keywords.
`"agreement" or "air" or "atmosphere" or "carbon" or "carbon dioxide" or "climate" or "climate change" or "co2" or "dioxide" or "earth" or "emissions" or "gas" or "global" or "greenhouse" or "heat" or "ice" or "land" or "nations" or "ocean" or "protocol" or "sea" or "solar" or "species" or "surface" or "temperature" or "warming"`

Fig 2. Number of articles collected for each COP meeting in the full dataset. COPs for which a larger share of articles was collected highlighted in orange.

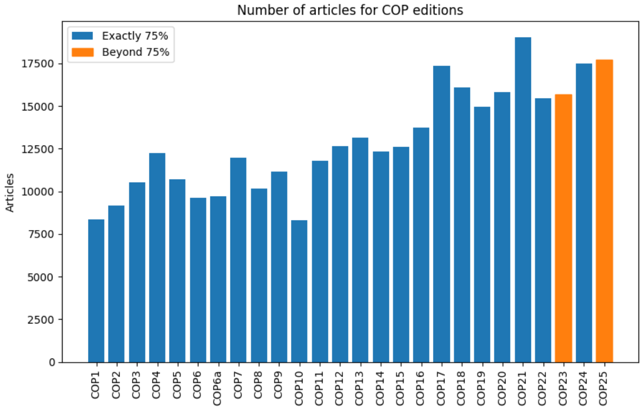

Fig 3. Number of articles for each COP meeting in the full dataset, before and after filtering for at least 3 unique keywords.

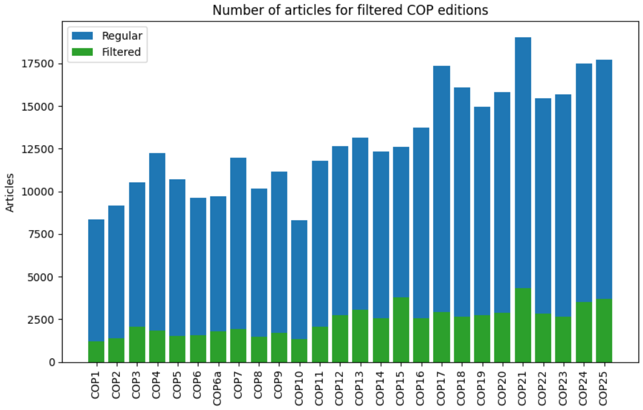

Fig 4. Number of absolute and unique keyword matches for COP3 articles in the full dataset, sorted by retrieval.

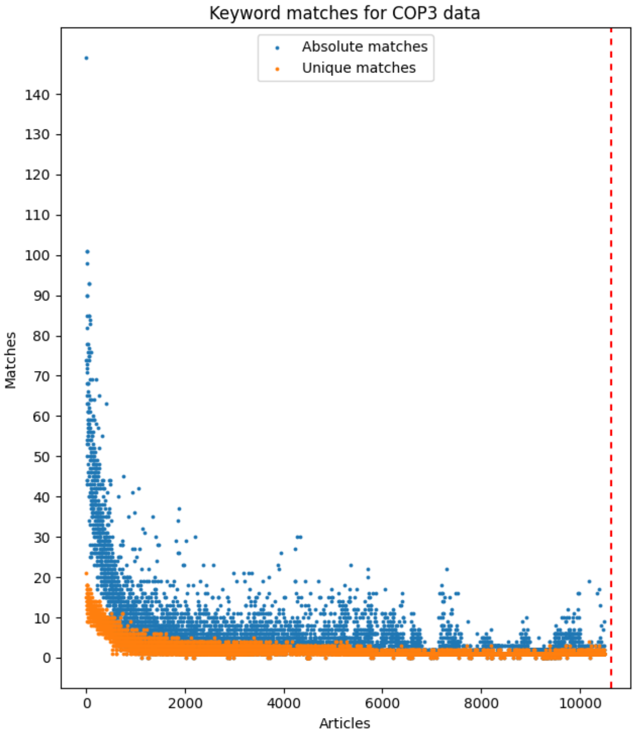

Fig 5. Number of unique keyword matches for COP3 articles in the full dataset, sorted by retrieval.

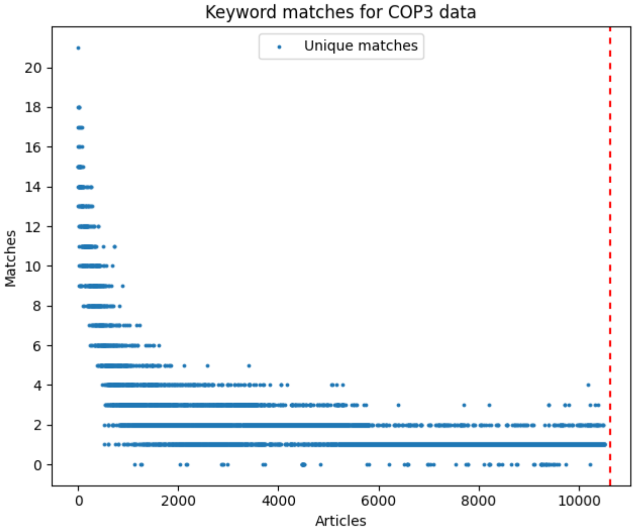

Supplement: S2 Appendix — Figures detailing the keyword selection process. (PDF) [file pone.0297644.s002.pdf]
