# Supplementary material for: Learning from climate change news: Is the world on the same page?
Source: PLoS One. 2024 Mar 20;19(3):e0297644. doi: 10.1371/journal.pone.0297644 (PMC10954114; doi:10.1371/journal.pone.0297644)
Supplement: S3 Appendix — Table of further statistics describing the data. (PDF) [file pone.0297644.s003.pdf]

Appendix 3: Data description

Table 7. Overview of the number of articles and their average length in the eventual dataset with three countries.

COP meeting	Number of articles	Avg. characters per article	Avg. words per article
COP1	861	5,327	888
COP2	940	5,712	953
COP3	1,553	5,192	865
COP4	1,318	5,524	921
COP5	1,036	6,547	1,091
COP6	1,082	5,654	944
COP6a	1,234	5,223	868
COP7	1,264	5,982	1,001
COP8	1,022	5,731	952
COP9	1,141	6,410	1,066
COP10	939	5,882	983
COP11	1,545	5,619	939
COP12	2,229	5,367	891
COP13	2,418	5,155	857
COP14	1,934	5,263	871
COP15	3,184	4,710	779
COP16	1,860	5,323	884
COP17	1,960	6,097	1,014
COP18	1,783	5,691	949
COP19	1,843	5,641	941
COP20	1,960	8,109	1,338
COP21	3,068	6,098	1,010
COP22	1,564	5,450	903
COP23	1,443	6,320	1,048
COP24	2,086	7,890	1,305
COP25	2,305	8,219	1,357
**Average**	**1,676**	**5,928**	**985**
**Total**	**43,572**	**154,136**	**25,618**
