# Supplementary material for: Learning from climate change news: Is the world on the same page?
Source: PLoS One. 2024 Mar 20;19(3):e0297644. doi: 10.1371/journal.pone.0297644 (PMC10954114; doi:10.1371/journal.pone.0297644)
Supplement: S4 Appendix — Further details on the feature selection process. (PDF) [file pone.0297644.s004.pdf]

Appendix 4: Feature selection

Overview of all 8 individual filtering steps applied during preprocessing of the data to prepare it for machine learning.

1. **Named entity removal**
   Each article was parsed using *SpaCy* (`spacy.io`) in order to recognize any named entities occurring in the text. The `en_core_web_md` (`spacy.io/models/en#en_core_web_md`) model was loaded for this. This model distinguishes 18 different named entity labels (`spacy.io/models/en#en_core_web_md-labels`). Recognized entities of all types were removed from the text. This step was intended to rigorously remove phrases like location and newspaper names. Some of the subsequent steps were thus used as a fallback, since *SpaCy* could detect some but not all entities flawlessly.
   Example: `United Nations`

2. **URL removal**
   All tokens that contained any of the domain names from the articles' newspapers were removed. This way, the entire URL or e-mail address was discarded. The same was done with strings containing the domain name extensions from the newspapers' countries. While this would likely already remove most overpredictive URLs, strings containing "*http*" or "*www*" were also filtered out, considering that no URLs were particularly of interest for the study.
   Example: `www.theaustralian.com.au/world`

3. **All-caps phrase removal**
   Phrases written entirely in capital letters were deleted as well. Typically, these were structure markers that were present specifically within some newspapers' articles and therefore had too much predictive power. Since it was hard to define all of these and since all-caps phrases could also represent abbreviated named entities, all of them were removed rigorously.
   Example: `ABSTRACT`

4. **COP meeting name removal**
   Since all articles were published in timeframes around COP meetings, it was highly likely that these were explicitly mentioned within the dataset. These references would be a direct mention of the corresponding edition label or number. All of them were hence removed.
   Example: `COP25`

5. **Newspaper name removal**
   The names of the included newspapers were also filtered out, again in order to remove features that could directly give away the related country or political orientation.
   Example: `The New York Times`

6. **Newspaper location removal**
   In line with newspapers typically being locally focused, it was expected that mentions of their country or city of origin occurred frequently. These could then easily give away from which country and possibly which orientation the article was. Any reference to an included newspaper's city, country or nationality was therefore deleted.
   Example: `Johannesburg`

7. **COP location removal**
   Like for the newspapers, the locations where COP meetings were held, could be overpredictive for the edition label itself. Hence, all names of cities, countries and nationalities that ever hosted a COP edition were removed.
   Example: `Kyoto`

8. **American English normalization**
   An intermediate training run of the model revealed that, with many other overpredictive features removed, there was one more aspect that could give away the country of publication. The variations between American and British orthography were picked up by the model. Hence, 1,722 American English words (`tysto.com/uk-us-spelling-list.html`) in the dataset were normalized to British English.
   Example: `airplanes` → `aeroplanes`
