# Supplementary material for: Learning from climate change news: Is the world on the same page?
Source: PLoS One. 2024 Mar 20;19(3):e0297644. doi: 10.1371/journal.pone.0297644 (PMC10954114; doi:10.1371/journal.pone.0297644)
Supplement: S5 Appendix — Tables detailing model experiments and the resulting performance. (PDF) [file pone.0297644.s005.pdf]

Appendix 5: Experiments & Performance

Table 8. Overview of accuracy and macro F1-scores for experiments conducted during classification model development.

Changes indicated in **bold**. Final model highlighted in gray.
(W=Word, C=Character, EM=Embeddings, B=Body, H=Headline)

#	Features	Preprocessing	COP		Country		Orientation	
			**Acc.**	**F1**	**Acc.**	**F1**	**Acc.**	**F1**
0	Baseline → Stratified `DummyClassifier`		0.04	0.04	0.34	0.33	0.51	0.50
1	**W1 (H+B)**	*None*	0.51	0.48	0.94	0.94	0.83	0.82
2	**W1-3 (H+B)**	*None*	0.54	0.50	0.93	0.93	0.83	0.82
3	W1-3 (H+B), **C2-5 (H+B)** (unweighted)	*None*	0.54	0.51	0.96	0.96	0.89	0.88
4	**W1-3 (H)**	*None*	0.32	0.28	0.71	0.69	0.71	0.70
5	**W1-3 (B)**	*None*	0.53	0.50	0.93	0.93	0.83	0.82
6	**W1 (B)**	*None*	0.52	0.48	0.93	0.93	0.81	0.81
7	W1 (B)	**Named Entities (sm)**	0.42	0.38	0.89	0.89	0.77	0.77
8	W1 (B)	**Named Entities (md)**	0.41	0.37	0.88	0.88	0.77	0.76
9	W1 (B)	Named Entities (md), **URLs (v1)**	0.41	0.37	0.88	0.88	0.77	0.76
10	W1 (B)	Named Entities (md), **URLs (v3)**, **All-caps**, **COPs**	0.40	0.37	0.87	0.87	0.76	0.75
11	W1 (B)	Named Entities (md), URLs (v3), All-caps, COPs, **Newspapers**, **NP locations**	0.41	0.37	0.87	0.87	0.76	0.75
12	**W1-3 (B)**	Named Entities (md), URLs (v3), All-caps, COPs, Newspapers, NP locations	0.43	0.39	0.87	0.87	0.78	0.77
13	W1-3 (B)	Named Entities (md), URLs (v3), All-caps, COPs, Newspapers, NP locations, **COP locations**, **English**	0.43	0.39	0.87	0.87	0.78	0.77
14	W1-3 (B)	Named Entities (md), **URLs (v4)**, All-caps, COPs, **Newspapers (v2)**, **NP locations (v2)**, COP locations, English	0.43	0.39	0.88	0.88	0.79	0.78
15	W1-3 (B)	Named Entities (md), **URLs (v5)**, All-caps, COPs, Newspapers (v2), NP locations (v2), COP locations, English (**Bugfix**)	0.43	0.39	0.87	0.87	0.78	0.77
16	W1-3 (B), **EM (H)**	Named Entities (md), URLs (v5), All-caps, COPs, Newspapers (v2), NP locations (v2), COP locations, English	0.44	0.40	0.87	0.87	0.79	0.77
17	W1-3 (B), **EM (H+B)**	Named Entities (md), URLs (v5), All-caps, COPs, Newspapers (v2), NP locations (v2), COP locations, English	0.43	0.39	0.90	0.90	0.80	0.79

Table 9. Overview of error scores for experiments conducted during regression model development.

Changes indicated in **bold**. Final model highlighted in gray.
(W=Word, C=Character, EM=Embeddings, B=Body, H=Headline)

#	Features	Preprocessing	MAE	MSE	RMSE
1	**W1 (B)**	*None*	3.37	21.75	4.66
2	**W1 (H+B)**	*None*	3.22	19.11	4.37
3	**W1-3 (B)**	*None*	2.72	14.11	3.76
4	**W1-3 (H+B)**	*None*	2.97	16.49	4.06
5	W1-3 (H+B)	**Named Entities (md), URLs (v5), All-caps, COPs, Newspapers (v2), NP locations (v2), COP locations, English**	3.39	20.44	4.52
6	**W1-3 (B)**	Named Entities (md), URLs (v5), All-caps, COPs, Newspapers (v2), NP locations (v2), COP locations, English	3.16	18.17	4.26
7	W1-3 (B), **C2-5 (B)**	Named Entities (md), URLs (v5), All-caps, COPs, Newspapers (v2), NP locations (v2), COP locations, English	3.16	18.17	4.26
8	W1-3 (B), **EM (H+B)**	Named Entities (md), URLs (v5), All-caps, COPs, Newspapers (v2), NP locations (v2), COP locations, English	2.72	14.00	3.74
